# Structural impact on SARS-CoV-2 spike protein by D614G substitution

**DOI:** 10.1126/science.abf2303

**Published:** 2021-03-16

**Authors:** Jun Zhang, Yongfei Cai, Tianshu Xiao, Jianming Lu, Hanqin Peng, Sarah M. Sterling, Richard M. Walsh, Sophia Rits-Volloch, Haisun Zhu, Alec N. Woosley, Wei Yang, Piotr Sliz, Bing Chen

**Affiliations:** 1 Division of Molecular Medicine, Boston Childrens Hospital, Boston, MA 02115, USA.; 2 Department of Pediatrics, Harvard Medical School, Boston, MA 02115, USA.; 3 Codex BioSolutions, Inc., Gaithersburg, MD 20879, USA.; 4 The Harvard Cryo-EM Center for Structural Biology, Harvard Medical School, Boston, MA 02115, USA.; 5 Department of Biological Chemistry and Molecular Pharmacology, Blavatnik Institute, Harvard Medical School, Boston, MA 02115, USA.; 6 Institute for Protein Innovation, Harvard Institutes of Medicine, Boston, MA 02115, USA.

## Abstract

Substitution for aspartic acid (D) by glycine (G) at position 614 in the spike (S) protein of severe acute respiratory syndrome coronavirus 2 (SARS-CoV-2) appears to facilitate rapid viral spread. The G614 strain and its recent variants are now the dominant circulating forms. Here, we report cryoelectron microscopy structures of a full-length G614 S trimer, which adopts three distinct prefusion conformations that differ primarily by the position of one receptor-binding domain. A loop disordered in the D614 S trimer wedges between domains within a protomer in the G614 spike. This added interaction appears to prevent premature dissociation of the G614 trimereffectively increasing the number of functional spikes and enhancing infectivityand to modulate structural rearrangements for membrane fusion. These findings extend our understanding of viral entry and suggest an improved immunogen for vaccine development.

## How an early variant got ahead

Throughout the COVID-19 pandemic, epidemiologists have monitored the evolution of severe acute respiratory syndrome coronavirus 2 (SARS-CoV-2) with particular focus on the spike protein. An early variant with an aspartic acid (D) to glycine (G) mutation at position 614, D614G, rapidly became dominant and is maintained in current variants of concern. Zhang *et al.* investigated the structural basis for the increased spread of this variant, which does so even though it binds less tightly to the host receptor (see the Perspective by Choe and Farzan). Structural and biochemical studies on a full-length G614 spike trimer showed that there are interactions not present in D614 that prevent premature loss of the S1 subunit that binds angiotensin-converting enzyme 2. This stabilization effectively increases the number of spikes that can facilitate infection.

*Science*, this issue p. 525; see also p. 466

## Teaser

A recently emerged variant prevents premature shedding of the host binding domain of the SARS-CoV-2 viral spike, which may increase infectivity.

Severe acute respiratory syndrome coronavirus 2 (SARS-CoV-2), an enveloped positive-stranded RNA virus, is the cause of the COVID-19 pandemic (*1*). Although the viral evolution is slowed by the RNA proofreading capability of its replication machinery (*2*), a variant with a single-residue substitution (D614G) in its spike (S) protein rapidly became the dominant strain throughout the world (*3*). It has since further evolved to give several variants of concern (VOCs) (*4*,*6*). The trimeric S protein decorates the viral surface and is an important target for the development of diagnostics, therapeutics, and vaccines; therefore, understanding the effect of key mutations may guide intervention strategies. Here, we focus on the D614G mutation that is in all currently circulating strains of SARS-CoV-2. The S protein is produced as a single-chain precursor and is subsequently processed by a furin-like protease into the receptor-binding fragment S1 and the fusion fragment S2 (*7*). After engagement of the receptor-binding domain (RBD) in S1 with the viral receptor angiotensin-converting enzyme 2 (ACE2) on the host cell surface, followed by a second proteolytic cleavage within S2 (S2 site) (*8*), the S protein undergoes large conformational changes, which result in the dissociation of S1 and the irreversible refolding of S2 into a postfusion structure (*9*, *10*). This induces fusion of the virus and host cell membranes to initiate infection. Rapid advances in structural biology of the SARS-CoV-2 S protein include structures of its soluble fragments: the ectodomain stabilized in its prefusion conformation (*11*,*13*), RBD-ACE2 complexes (*14*,*17*), and segments of S2 in the postfusion state (*18*). In the prefusion ectodomain structure, S1 folds into four domainsthe N-terminal domain (NTD), the RBD, and two C-terminal domains (CTDs)and wraps around the prefusion S2, with the RBD sampling two distinct conformations: up for a receptor-accessible state and down for a receptor-inaccessible state. We and others have reported structures of a purified, full-length D614 S protein in both prefusion and postfusion conformations (*19*, *20*). Studies by cryoelectron tomography (cryo-ET), with chemically inactivated SARS-CoV-2 preparations, using both D614 and G614 variants have revealed additional structural details of S proteins present on the surface of the virion (*21*,*24*).

Epidemiological surveillance has indicated that the SARS-CoV-2 carrying G614 outcompeted the original virus and became the globally dominant form within a month (*3*, *25*, *26*). This single-residue substitution appears to correlate with high viral loads in infected patients and high infectivity of pseudotyped viruses, but not with disease severity (*3*). The G614 virus has comparable sensitivity to neutralization by convalescent human sera or vaccinated hamster sera (*3*, *27*,*30*), which suggests that vaccines containing D614 remain effective against the G614 virus. Moreover, S1 dissociates more readily from the D614 virus than from G614 virus (*31*), which indicates that the D614 viral spike is less stable than that of the G614 variant. The G614 ectodomain trimer is reported to sample the RBD-up conformations more frequently than the D614 trimer (*13*, *29*, *32*), but it is puzzling why the former binds more weakly to recombinant ACE2 than the latter (*32*). The known S trimer structures indicate that the D614G change breaks a salt bridge between D614 and a lysine residue (K854) in the fusion peptideproximal region (FPPR) (*19*, *33*, *34*), which may help clamp the RBD in the prefusion conformation. This observation can explain why the G614 trimer favors the RBD-up conformations but does not account for its increased stability. To resolve these issues, we report the structural consequences of the D614G substitution in the context of the full-length S protein.

**Fig. 1:**
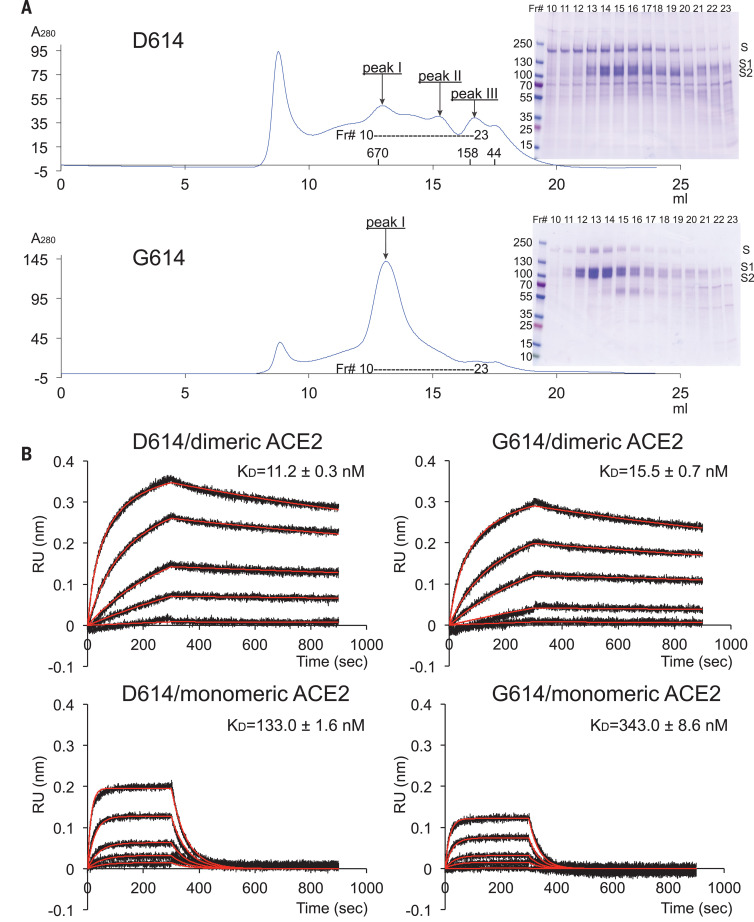
Characterization of the purified full-length SARS-CoV-2 S proteins. (**A**) The full-length SARS-CoV-2 S protein carrying either D614 or G614 was extracted and purified in detergent n-dodecyl--d-maltopyranoside (DDM) and further resolved by gel-filtration chromatography on a Superose 6 column. The molecular weight standards include thyoglobulin (670 kDa), -globulin (158 kDa), and ovalbumin (44 kDa). Peak I is the prefusion S trimer; peak II is the postfusion S2 trimer, and peak III is the dissociated monomeric S1. The insets show peak fractions that were analyzed by Coomassie-stained SDS-PAGE. Labeled bands are S, S1, and S2. Fr#, fraction number. (**B**) Binding analysis of fractions of peak I in (A) with soluble ACE2 constructs by BLI. The purified S proteins were immobilized to AR2G biosensors and dipped into the wells containing ACE2 at various concentrations (5.56 to 450 nM for monomeric ACE2; 2.78 to 225 nM for dimeric ACE2). Binding kinetics was evaluated using a 1:1 Langmuir binding model for the monomeric ACE2 and a bivalent model for dimeric ACE2. The sensorgrams are in black and the fits in red. Binding constants are also summarized here and in table S1. All experiments were repeated at least twice with essentially identical results. K_D_, dissociation constant (binding affinity); RU, response unit.

We compared the membrane fusion activity of the full-length G614 S protein (fig. S1) with that of the D614 S construct in a cell-cell fusion assay (*19*). All of the cells expressing S fused efficiently with cells transfected with a human ACE2 construct (fig. S2A), demonstrating that the S proteins expressed on the cell surfaces are fully functional. At low transfection levels, G614 S had higher fusion activity than the D614 S, but the difference diminished with the increased amount of transfected DNA, which suggests that the high expression levels can compensate for lower fusion efficiency of the D614 S protein. The G614 trimer remains sensitive to inhibition by an engineered trimeric ACE2-based inhibitor that competes with the receptor on the target cells (*35*) (fig. S2B). For protein purification, we used a construct fused with a C-terminal strep-tag, which was equally active in cell-cell fusion as the untagged version (fig. S2A), and purified both G614 and D614 proteins under identical conditions. The D614 protein eluted in three peaks characterized previously as the prefusion S trimer, the postfusion S2 trimer, and the dissociated monomeric S1 (*19*). The G614 protein eluted as a single major peak, corresponding to the prefusion trimer (Fig. 1A). This suggests that D614G has a notable effect on the stability of the SARS-CoV-2 S trimer. Coomassie-stained SDSpolyacrylamide gel electrophoresis (SDS-PAGE) analysis shows that G614 elutes mainly as the prefusion trimer, comprising the cleaved S1-S2 complex (~90%) and a small amount of the uncleaved S precursor (~10%). We next measured binding of the prefusion trimer fractions of the full-length proteins to recombinant soluble ACE2 by biolayer interferometry (BLI) (Fig. 1B). The S trimers bound more strongly to a dimeric ACE2 than to a monomeric ACE2, as expected. The G614 protein bound ACE2 less tightly than the D614 protein, which is consistent with the measurements reported by others using soluble constructs (*32*). This observation appears inconsistent with accounts that the G614 trimer has a more exposed RBD than the D614 trimer (*13*, *21*, *22*, *29*, *32*). We note that the second binding event between dimeric ACE2 and a G614 trimer has both a slower on-rate and a slower off-rate than that for a D614 trimer (table S1).

**Fig. 2:**
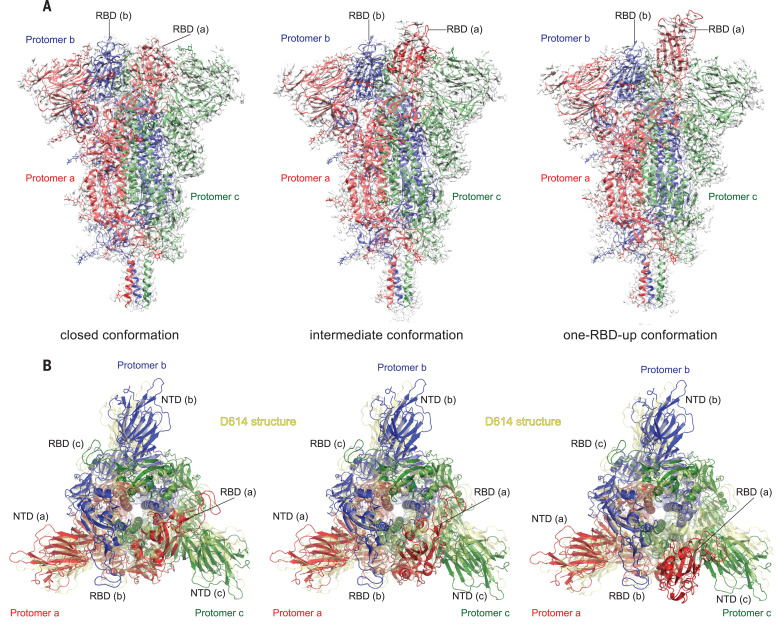
Cryo-EM structures of the full-length SARS-CoV-2 S protein carrying G614. (**A**) Three structures of the G614 S trimerrepresenting a closed, three RBDdown conformation; an RBD-intermediate conformation; and a one RBDup conformationwere modeled on the basis of corresponding cryo-EM density maps at 3.1- to 3.5- resolution. Three protomers (a, b, and c) are colored in red, blue, and green, respectively. RBD locations are indicated. (**B**) Top views of the superposition of the three structures of the G614 S in (A) in ribbon representation, with the structure of the prefusion trimer of the D614 S (Protein Data Bank ID: 6XR8) shown in yellow. The NTD and RBD of each protomer are indicated. Side views of the superposition are shown in fig. S8.

We determined the cryoelectron microscopy (cryo-EM) structures of the full-length G614 S trimer using RELION (*36*). Three-dimensional (3D) classification identified three distinct classes, each containing a similar number of particles. The three classes represent a closed, three RBDdown conformation; a one RBDup conformation; and an intermediate conformation with one RBD flipped up only halfway. All structures were refined to 3.1- to 3.5- resolution (figs. S3 to S8 and table S2). The overall structure of the G614 S protein in the closed, three RBDdown prefusion conformation is very similar to that of our published D614 S trimer (Fig. 2) (*19*). In the three RBDdown structure, the four domains in each S1, including NTD, RBD, CTD1, and CTD2, wrap around the three-fold axis of the trimer, protecting the prefusion S2. The furin cleavage site is disordered, which makes it uncertain whether this structure represents the uncleaved or cleaved trimer, although the preparation contains primarily the cleaved forms (Fig. 1A). The S2 fragment folds around a central three-stranded coiled coil that forms the most stable part of the structure; it is also the least variable region among all of the known S trimer structures. The S2 structure is identical in the two G614 structures, with one RBD projecting upward, either completely or partially (fig. S9). In the conformation with one RBD fully up, the two neighboring NTDs, including the one from the same protomer, shift away from the three-fold axis (fig. S9). In the RBD-intermediate conformation, only the NTD from the adjacent protomer packing directly against the moving RBD shifts. The D614G substitution eliminates a salt bridge between D614 in CTD2 of one subunit and K854 in the FPPR of the adjacent subunit (*19*, *34*), but the FPPR in the three RBDdown conformation of the G614 trimer remains structured.

**Fig. 3:**
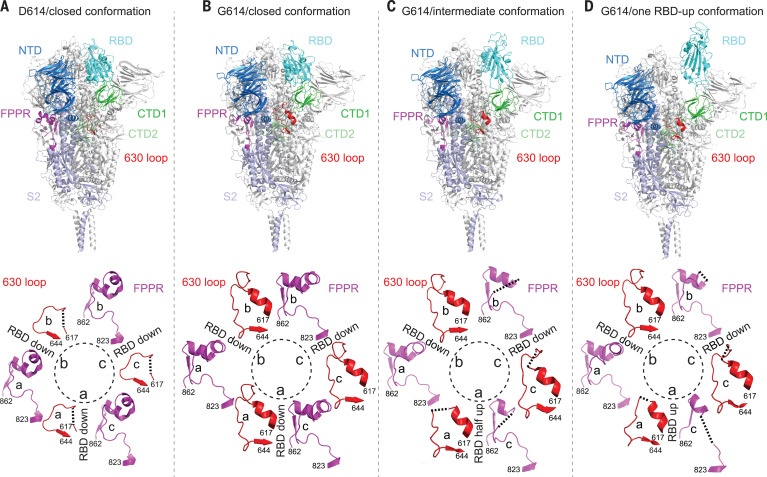
Cryo-EM structures of the full-length SARS-CoV-2 S protein carrying G614. (**A**) (Top) The structure of the closed, three RBDdown conformation of the D614 S trimer is shown in ribbon diagram with one protomer colored as NTD in blue, RBD in cyan, CTD1 in green, CTD2 in light green, S2 in light blue, the 630 loop in red, and the FPPR in magenta. (Bottom) Structures of three segments (residues 617 to 644) containing the 630 loop in red and three segments (residues 823 to 862) containing the FPPR in magenta from all three protomers (a, b, and c) are shown. The position of each RBD is indicated. (**B** to **D**) Structures of the G614 trimer in the closed, three RBDdown conformation, the RBD-intermediate conformation, and the one RBDup conformation, respectively, are shown, as in (A). Dashed lines indicate gaps.

**Fig. 4:**
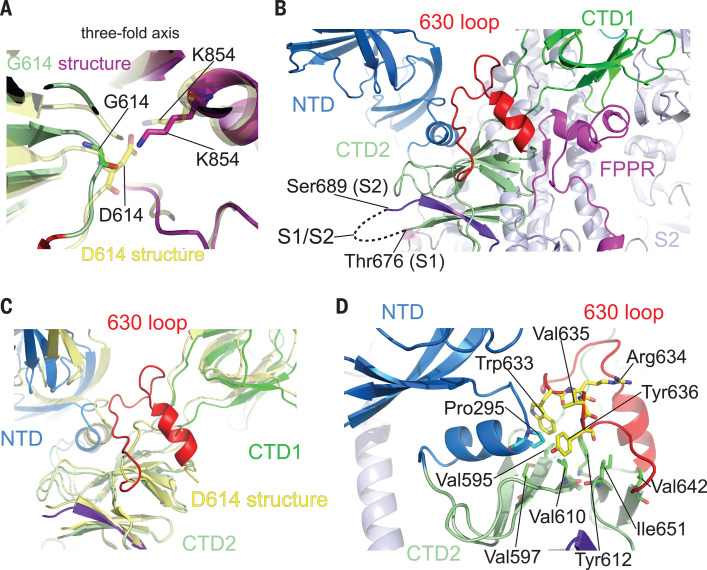
Close-up views of the D614G substitution. (**A**) A close-up view of the region near the residue 614 with superposition of the G614 trimer structure in green (CTD2) and magenta (FPPR) and the D614 trimer in yellow, both in the closed prefusion conformation. Residues G614, D614, and two K854s from both structures are shown in stick model. The direction of the three-fold axis of the trimer is indicated. (**B**) Location of the 630 loop in the S trimer. The 630 loop is highlighted in red, the NTD in blue, the CTD1 in green, the CTD2 in light green, the S2 in light blue, and the FPPR from a neighboring protomer in magenta. The S1-S2 boundary and the nearest ordered residues Thr^676^ from S1 and Ser^689^ from S2 are all indicated. A strand from the N-terminal end of S2, packed in the CTD2, is highlighted in purple. (**C**) A view showing that the 630 loop wedges between the NTD and the CTD1 and pushes them apart. (**D**) Packing of the 630 loop against the hydrophobic surface formed by residues Val^595^, Val^597^, Val^610^, Tyr^612^, Val^642^, and Ile^651^ from the CTD2 and Pro^295^ from the NTD. Residues Trp^633^ and Val^635^ from the 630 loop contribute to this interaction.

To examine the structural changes resulting from the D614G substitution, we superposed the structures of the G614 trimer onto the D614 trimer in the closed conformation, aligning them by the invariant S2 (Fig. 2B). A shift by a clockwise, outward rotation of all three S1 subunits, relative to the D614 structure, is evident even for the G614 trimer in the closed conformation. A similar shift was also observed in the RBD-intermediate and RBD-up G614 structures. Thus, the D614G substitution has led to a slightly more open conformation than that of the D614 trimer, even when all three RBDs are down. The D614G change has apparently also rigidified a neighboring segment of CTD2, residues 620 to 640, which we designate the 630 loop. This loop inserts into a gap, slightly wider in the G614 than in the D614 trimer, between the NTD and CTD1 of the same protomer (Figs. 3 and 4). The 630 loop is disordered in the closed D614 trimer (fig. S10) because the gap is too narrow for it to insert. The closed D614 trimer thus has three ordered FPPRs and three disordered 630 loops, whereas the closed G614 trimer has three structured 630 loops along with three ordered FPPRs. In the two conformers with one partly or fully open RBD, the two segments are disordered in the RBD-shifted subunit, and their central parts have difficult-to-model densities in one other subunit. The third pair appears well ordered throughout (Fig. 3). Thus, the opening of the RBD in the full-length G614 trimer correlates with a displacement of the 630 loop and the FPPR having moved away from its position in the D614 trimer.

The D614G change did not cause any large local structural rearrangements except for loss of the D614-K854 salt bridge (*19*, *34*) and a small shift of residue 614 toward the three-fold axis (Fig. 4A). The position of the FPPR and the conformation of K854 may allow a hydrogen bond between the K854 amino group and the main-chain carbonyl of G614, perhaps accounting for the subtlety of the structural difference (fig. S11, A and B). Although the loss of the salt bridge involving D614 does not destabilize the packing of the FPPR against the rest of the trimer, it does weaken the FPPR density, especially between residues 842 and 846. The 630 loop, which packs directly against the NTD, CTD1, and CTD2 of the same protomer, lies close to the S1-S2 boundary of the same protomer and the FPPR of an adjacent protomer (Fig. 4B). Inserting this wedge-like loop between the NTD and CTD1 (Fig. 4C) may help secure the positions of the NTD and CTDs.

CTD2 is formed by two stacked, four-stranded sheets, with a fifth strand in one sheet contributed by the connector between the NTD and RBD. In the other sheet, an interstrand loop contains the S1-S2 cleavage site, and thus one strand is the N-terminal segment of S2 (Fig. 4B). In the G614 trimer, one side of the 630 loop packs along a long hydrophobic surface, largely solvent-exposed in the D614 trimer, formed by residues on the upward-facing surface of the CTD2 along with Pro^295^ from the NTD (Fig. 4D). Trp^633^ and Tyr^636^ of the 630 loop appear to contribute to this interaction. S1 dissociation from S2 requires breaking the S2 strand from the second sheet. An ordered 630 loop that stabilizes the CTD2 by closing off an exposed, hydrophobic surface may retard S1 shedding, thereby enhancing the stability of a cleaved S trimer. We note that the density for a fatty acid ligand making contacts with the neighboring RBDs in the D614 trimer is absent in all of the G614 reconstructions (fig. S11C) (*37*), which suggests that the ligand is not required for three RBDs to adopt the down conformation.

To test the impact of the 630 loop on S1 shedding and membrane fusion, we generated five S mutants, each containing a single-residue change either in the 630 loop (W633A, R634E, V635K, and Y636A) or the CTD2 hydrophobic surface (V610K) in the G614 sequence. These mutants expressed the same levels of S, with similar extents of cleavage between S1 and S2, as expected (fig. S12A). When detected by monoclonal antibodies using flow cytometry, mutants V610K and W633A showed markedly lower binding of RBD-specific antibodies [REGN10933 and REGN10987; (*38*)] and of an NTD-specific antibody [4A8; (*39*)] than the parental G614 S, whereas binding to an S2-specific antibody [0304-3H3; (*39*)] was slightly higher (fig. S12B). These results are consistent with the hydrophobic interactions between the 630 loop and CTD2 stabilizing the cleaved S1-S2 complex and preventing S1 dissociation. The mutant V635K had wild-type phenotypes in these assays, likely because V635 does not make any direct contact with the CTD2. The mutants R634E and Y636A showed intermediate levels of antibody binding because Y636 appears to contribute less to the 630 loopCTD2 interaction than W633, and R634 may help maintain the loops overall shape for inserting between domains. Likewise, a similar pattern was observed with these mutants in the cell-cell fusion assay, except that Y636A showed substantially weaker fusion activity than R634E (fig. S12C). Thus, key residues important for stabilizing the S trimer structure appear critical for membrane fusion activity, as premature dissociation of S1 would lead to inactivation of the S trimer.

To further confirm the folding of the 630 loop in the G614 trimer, we collected additional data under slightly different conditions and found the same three classes representing the closed, RBD-intermediate, and one RBDup conformations (fig. S13A). There is relatively strong density in both 2D class averages and 3D reconstructions for the heptad repeat 2 (HR2) region (fig. S1) and detergent micelle, which were invisible in all our previous cryo-EM analyses. The increased length and lack of symmetry limited the resolution of these 3D reconstructions to 4.3 to 4.7 . Nevertheless, the density for the 630 loop was evident in the closed trimer even at this resolution (fig. S13B). We note that although S1 in the G614 trimer moves outward from its position in the D614 trimer, the extent of the shift is still appreciably smaller than the shift seen in soluble S trimers stabilized by a trimerization foldon tag and two proline mutations (fig. S14).

Our structures provide an explanation for why the G614 virus, with a more stable S trimer, is more infectious than the original strain (fig. S15). The transition from the closed to the one RBDup conformation in a G614 trimer requires an order-disorder transition in one 630 loop and a partial disordering of a second. Thus, kinetic barriers will probably make both the forward and reverse transitions slower than in a D614 trimer, in which all three 630 loops are unstructured in both conformations. In the one RBDup conformation, S will also shed S1 much more slowly from a G614 trimer than from D614 because the remaining two RBDs are stabilized by the ordered and partially ordered 630 loops, and a return of the first RBD to the down configuration can occur unless locked in place by ACE2 receptor binding. This can account for both the greater prevalence of a one RBDup conformation and a lower overall ACE2 affinity, because the other two RBDs will remain inaccessible. It can also explain why we captured very few trimers in the RBD-up conformation in our previous cryo-EM study of the D614 trimer but instead saw abundant postfusion S2 (*19*), because any one RBDup conformation would proceed to two RBDup and three RBDup and shed very quickly, allowing S2 to convert to the postfusion form.

Our interpretation of the structural differences is also consistent with the spike conformational distribution on the virions in cryo-ET studies of chemically inactivated SARS-CoV-2. The D614 preparation contains primarily postfusion S2 spikes (*24*). One study of a G614 virus that had lost the furin cleavage site showed almost no postfusion spikes and a 50:50 distribution of prefusion spikes between fully closed and one RBDup (*21*), and another showed 3% postfusion spikes and 97% in the prefusion form (~31%, fully closed; ~55%, one RBDup; and ~14%, two RBDup) (*22*). The structured 630 loop in the G614 trimer not only reinforces the packing among three protomers but also stabilizes the CTD2 to inhibit release of the N-terminal segment of S2, effectively blocking S1 dissociation. This property can account for the paucity of postfusion spikes on the G614 variant.

In addition to the FPPR that might modulate the fusogenic structural rearrangements of S protein (*19*), CTD2 and the 630 loop within it are probably also the key components of the S fusion machinery. If ACE2 captures the RBD-up conformation (*40*), expelling both the 630 loop and the FPPR from their positions in the closed S trimer conformation, the FPPR shift may help expose the S2 site near the fusion peptide for proteolytic cleavage, whereas departure of the 630 loop from the hydrophobic surface of the CTD2 can destabilize this domain and free the N-terminal segment of S2 to dissociate from S1if the furin site has already been cleavedand release S1 altogether. Dissociation of S1 would then initiate a cascade of refolding events in the metastable prefusion S2, allowing the fusogenic transition to a stable postfusion structure. This model is similar to that proposed for membrane fusion catalyzed by HIV envelope protein (*41*).

The SARS-CoV-2 S protein is the centerpiece of the first-generation vaccines that almost all used the D614 sequence. The G614 S trimer is naturally constrained in a prefusion state that presents both the RBD-down and RBD-up conformations with great stability. It is therefore likely to be a superior immunogen for eliciting protective neutralizing antibody responses, which appear to largely target the RBD and NTD (*39*, *42*). It may also be an excellent scaffold for designing next-generation vaccines against new variants that have become resistant to the protections offered by the existing vaccines (*43*,*46*).

We suggest that the enhanced infectivity of the G614 virus largely results from the increased stability of the S trimer, rather than the better-exposed RBDs. If the virus that passed from bats to humans, or to an intermediate vector, contained D614 [also present in the bat coronavirus, BatCoV RaTG13 (*1*)], then it could have gained fitness in the new host by acquiring changes such as G614 for greater stability and infectivity than that observed in the parental form. Unsurprisingly, the recent fast-spreading variantsincluding the B.1.1.7 (VUI202012/01; 501Y.V1) lineage from the United Kingdom, the B.1.351 (501Y.V2) lineage from South Africa, and the B.1.1.28 (484K.V2; P.1) lineage from Brazil (*4*,*6*)all contain the D614G substitution (table S3), which suggests that the increased transmissibility of the G614 virus has led to a great number of replication events and to greater genetic diversity, despite a lower absolute mutation rate.

## Supplementary Materials

science.sciencemag.org/content/372/6541/525/suppl/DC1 Materials and Methods Figs. S1 to S15 Tables S1 to S3 References (*47*,*56*) MDAR Reproducibility Checklist

## Appendix

View/request a protocol for this paper from *Bio-protocol*.
